# Basic assessment of microbial dynamics in large- and small-scale biofilters

**DOI:** 10.1016/j.btre.2025.e00936

**Published:** 2025-11-04

**Authors:** Andreas Otto Wagner, Julia Wurm, Mathias Wunderer, Julia Zöhrer, Andja Mullaymeri, Eva-Maria Weinseisen, Eva Maria Prem

**Affiliations:** a Department of Microbiology, Universität Innsbruck, Technikerstrasse 25d, 6020 Innsbruck, Austria; b Abfallbeseitigungsverband- Westtirol, Tschirgantweg 1, 6426 Roppen, Austria

**Keywords:** Biogas, Biofilters, Methanotrophy, Odour reduction, Amplicon sequencing

## Abstract

Treating exhaust gas from biogas plants is commonly done using biofilters composed of different filter materials. Immobilized on these filter materials microbes can convert less desirable exhaust gas components into more wanted ones. The evaluation of the performance of those filters based on microbial data is, however, difficult due to a lack in data regarding microbial colonisation of these filter materials. Thus, in the present study microbial abundance and microbial community composition in large- and small-scale biofilters was evaluated using both, cultivation dependent and independent approaches. Large-scale biofilters showed a similar total abundance of bacteria irrespective of the filter material and the water content of the biofilter. In contrast, fungal and yeast abundance was impacted by both, filter material (bark mulch or coconut fibre) and water content. In small scale biofilters (composed of bark mulch) the water content impacted microbial abundance. While a water content of 90% led to a similar development of bacterial and fungal/yeast abundance, 70% water content caused an asynchronous increase in abundance. Analysis of small-scale biofilters revealed an acidotrophic bacterial community including potential methanotrophs.

## Highlights

• Data on microbial abundance and community composition of biofilters
• Biofilters dominated by an acidophilic prokaryotic community
• Water content and biofilter material are impacting microbial colonisation

## Graphical abstract

**Figure:**
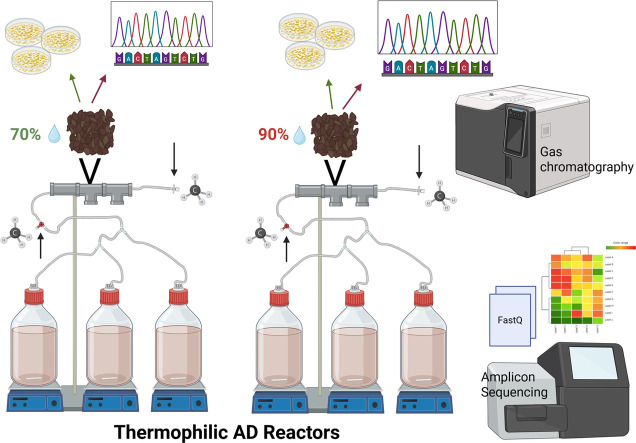

## 1 Introduction

Biofilters can be used in various systems for cleaning raw gases that contain different odorous substances [[1], [2], [3], [4]], provided, that exhaust air components are biologically degradable and water soluble [5]. Therefore, the function of biofilters is provided by the transfer of water-soluble compounds from the exhaust air into a water film to the biofilter material, where it can be subjected to biochemical interaction such as ad- and absorption and complexation and microbial attack. In contrast to non-biological technologies like thermal oxidation, photocatalytic oxidization, incineration, condensation and adsorption [6,7], the former often bear the advantage of lower energy input needed [8]. In addition, gas-phase biofilters were shown to be efficient in reducing bacterial emissions [9]. Organic biofilter materials include bark products (e.g. bark mulch, chipping, pellets), coconut wool/fibre, compost, root wood, heather, peat products (e.g. fibrous peat), while examples for inorganic filter materials comprise clay, lava material, as well as inorganic granulated and fibrous material [[10], [11], [12]]. The performance of biofilters depends on various factors, such as gas composition, retention time of gases to be cleaned and the environmental conditions within the filter; another important factor is the microbiological composition of the filter material [12,13]. This microbial consortium must be continuously supplied with substrate, water and gas [13]. These components are used by microbes to gain energy and to build up cell biomass [14] – this is accompanied by degradation of biofilter materials and thus regular replacement of those materials [13].

Microorganisms in biofilters are (one of) the main drivers of the conversion of the raw gas into other, more desirable compounds, which is why the composition of its microbial community and abundance plays a decisive role in estimating the performance of biofilters. Pollutants are broken down by the microorganisms living on and from the carrier material, which is providing the necessary matrix (water, pH, temperature), carbon, nitrogen and phosphorous supply (other to that, included in the exhaust gas mixture), mineral salts, and trace elements [5,13,15].This microbial biocenosis colonising surfaces and/or pores of the filter material mainly comprises of bacteria, archaea, fungi, and yeast [11]. While those growing on solid materials are better protected against changing conditions (pH value, drying out, temperature fluctuations) microorganisms in suspension are more susceptible against environmental changes [11]. However, data are rare on microbial composition and abundances on biofilter materials [11]. The genus *Pseudomonads* and coryneform bacteria were reported to be prevalent on biofilter materials, *Pseudomonas* sp. was also found to be important in methanol degrading biofilters [16], *Streptomyces griseus* was used for toluene degradation in an biofiltration process [7] and also *Ochrobactrum* spp. and *Klebsiella oxytoca* were found in methanol degrading biofilters [16]. The microbial diversity in a biofilter using compost as a matrix was investigated in another study [17]. In Austria, when undesired odours occur or when mandatory biofilter tests are required, the performance of biofilters is commonly assessed by olfactory estimations conducted by specially trained and qualified personnel (personal communication biogas plant operators). These tests are expensive and can be tricky because of difficulties during gas sampling, gas sample handling, conservation and transportation. As the microbiota in biofilters is hold responsible for the removal of odour nuisance, more data on the number, distribution and physiological potential of microbes within the biofilter material might help to better understand problems regarding biofilter performance.

Therefore, the aim of the present study was to provide data on microbial abundance and microbial community composition of biofilters using both, cultivation dependent and independent approaches to better estimate the performance of biofilters with respect to microbial colonialization of biofilter materials.

## 2 Material and methods

### 2.1 Experimental setup

The investigation was split into two parts. Biogas plant operators in Roppen (ABV Westtirol), Austria reported odour nuisance which indicated restricted performance of large-scale biofilters. Therefore, in a first approach, we sampled material from 3 different large-scale biofilters running at the biogas plant to assess biochemical characteristics and to quantify the cultivable microbial community via agar plating. For details regarding the running parameters of the biogas plant in Roppen, please refer to previous publications [18]. In a second approach, small-scale biofilters were established in the lab to investigate biochemical characteristics as well as the respective microbiome using a culture-based (agar plating) as well as a 16S V4 amplicon sequencing approach. While large-scale biofilters were fed with biogas from the large-scale digestion plant, small-scale filters consumed gas from a lab fermentation system (5 L) operated at thermophilic conditions according to the setup of [19].

### 2.2 Biofilters

**Fig. 1:**
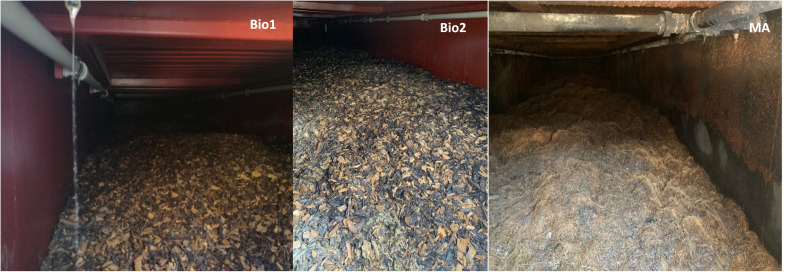
Biofilter material Biokamin 1 (Bio1), Biokamin 2 (Bio2) and MA with bark mulch (Bio1, Bio2) and coconut fiber (MA), respectively, shown within the housing. Irrigation pipes installed on the left- and right-hand side of the housing.

Large-scale filters were filled with bark mulch with a water content of 95.5 g/100 g (Biokamin 1, Bio1) and 30.4 g/100g (Biokamin 2, Bio2), or coconut fibre with a water content of 89.3 g/100 g (MA). More details on the large-scale biofilters are given in Table 1 and Fig. 1.

**Table 1:** Characteristics of large-scale biofilters. Parameters (water content, temperature, pH, H_2_S, ammonia, NO_3_^-^) were evaluated over a period of 2 years, between 2017 and 2019 (twice or 4-times a year if not otherwise indicated).

	Biokamin1	Biokamin2	MA
Housing	40 ft container	40ft container	30ft container
Kind of compound removal	Biological activity		
Filter material	Bark mulch	Bark mulch	Coconut fibre
Filter particle size	20 – 40 mm	20 – 40 mm	na*
Operating pressure	1 000 – max. 2 500 Pa		
Desired operating temperature	> +5°C; < + 35°C		
Gas-washing module for gas humidification	yes	yes	no
Raw gas water content [%] low- high (continuous online measurement)	76.6 - 100	84.7 - 100	na*
Filter material humidity control	Yes, automatic dosing		
Raw gas temperature [°C] low – high (continuous online measurement)	7.1 – 25.0	8.6 – 30.2	na*
pH (in aqua dest.) low - high	6.0 – 6.28	6.0 – 7.25	6.51 – 7.54
H_2_S effluent [ppm]	< 0.5	< 0.5	< 0.5
Ammonia effluent [ppm] low - high	38 - 50	30 - 38	15 - 20
NO_3_^-^ (filter material) [mg kg^-1^] low - high	10 - 50	10	10 - 500

*na: data not available

**Fig. 2:**
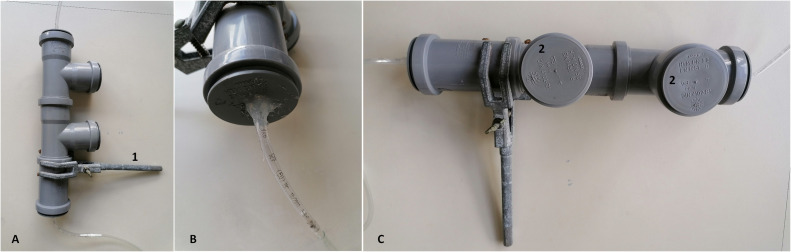
Small-scale biofilters made from PP (DIN 4102/B1) including a positioning part to keep the biofilter in an upright position via conventional laboratory holders (A, 1), in- and outlet-port (B) and sampling ports with removable caps (C, 2).

Small-scale biofilters were self-constructed (Fig. 2): Polypropylene (PP, DIN 4102/B1) pipes (Ostendorf HT DN 50) were used including a T-piece for sampling (Fig. 2C). Apertures at the top and bottom were closed with PP caps featuring gastight hose connections for gas in- and efflux tubes (5 mm ID), respectively. The T-piece was closed with a PP cap without additional modifications. Small-scale filters were filled with 55 g (DW) of bark mulch (Kranzinger, Austria; sieved to 20 – 40 mm) and moistened with double-autoclaved distilled water to reach water content of 70% w/w (BF1) and 90% w/w (BF2). For each biofilter, influx tubes were connected to 3 out of 6 parallel lab fermentation systems [19] operated with 2.0 g/L*day organic loading rate (mainly starch) and producing 600 – 700 NmL CH_4_ per day. The biogas derived from lab fermenters was not dried, so that water vapour was constantly entering the biofilters and those did not run dry. The upper level of bark mulch was reached approx. 1 cm below the biofilter cap but settled down during the experiments.

### 2.3 Sampling

During reactor maintenance, material from large-scale biofilters (Fig. 1) was taken from the upper 40 cm of the filter layer in April 2021. Approx. 1 kg of material of each filter (Biokamin1, Biokamin2, MA) was taken, filled into screw-cap bottles, transferred to the lab at ambient temperature (this took two hours), and analysed immediately. Biofilter material from coconut fibre was homogenized (fibre length <1 cm) using surface disinfected scissors. Material was stored for 10 days at 4°C and subsequently frozen (-20°C).

Small-scale reactors were sampled using the sampling port (Fig. 2). 1 g of each sample were taken from the middle of the filter at days 0, 7, 14, 21, 28, 34, 42, and 64.

### 2.4 Olfactory evaluation

To get an indication of the performance of the biofilters to reduce odour nuisance, 50 mL of untreated reactor headspace gas as well as 50 mL filtered gas were each added to a 5 L plastic bag using a 50 mL syringe (2 biofilters, n=4). In a blind test, those bags were presented to 6 colleagues separately (not engaged in the present investigation), opened for 3 seconds, and then assessed on a scale from 0 (no bad smell) to 5 (extremely bad smell).

### 2.5 Microbial, biochemical and gas analyses

All analyses were carried out in triplicate. For dry weight (DW) determination, 10 g (large-scale) and 2.5 g (small-scale) biofilter material or 10 g fresh bark mulch was dried overnight at 105°C and analysed for weight loss [g 100 g^-1^]. Analysis of colony forming units (cfu) was conducted from 1:10 diluted extracts of biofilter materials (small and large-scale) or fresh bark mulch prepared in ¼ Ringer solution (Ringer solution tablets, VWR, Germany). Solutions were shaken for 15 min at 25°C and 125 rpm, then further diluted 1:10 in ¼ Ringer solution (total volume of 10 mL for each dilution step) and 50 µl were applied to agar plates. Different agar media were tested (data not shown), whereby standard-1-agar for prokaryotic microorganisms (ST1: casein peptone 15.6 g, yeast extract 3.0 g, NaCl 6.0 g, glucose monohydrate 1.0 g, agar 20 g, 1 000 ml distilled water, pH 7.0, Cycloheximide 100 mg) and malt-extract-agar for fungi and yeast (MEA: malt extract 30 g, casein peptone 5.0 g, agar 20 g, distilled water 1000 ml, pH 6.8, Chloramphenicol 100 mg) turned out to provide the highest cfu-counts and thus used in further experiments. After spreading (diluted) extracts on the agar plates, those were incubated at 25°C for 72 h. Dilution stages resulting in 10 – 100 cfu per agar plate were used for quantitative analyses via cfu counting (number of cfu) and calculation according to formula 1

$$cfu\;per\;mL\;\left[cfu\;mL-1\right]=\frac{number\;of\;cfu}{0.05}*\frac{1}{dilution}$$

and formula 2

$$cfu\;per\;DW\;\left[cfu\;g\left(DW\right)-1\right]=\frac{cfu\;per\;mL}{DW}*100$$

whereby

*cfu per mL*: number of cfu per mL of extract [cfu mL^-1^]

*cfu per DW*: number of cfu per g of dry weight (DW) [cfu g^-1^]

*number of cfu*: plate count on respective dilution stage [cfu plate^-1^]

*dilution*: dilution stage (e.g., 1:10 = 10^-1^)

*DW*: dry weight [g 100 g^-1^]

To evaluate the capability of the small-scale biofilters to remove CH_4_ from the lab biogas reactor, biofilter in- and outlet gas samples were analysed using a Shimadzu GC-2010 gas chromatograph equipped with a FID@170°C and TCD@180°C and a ShinCarbon column 100/120 mesh (2 m, Restek, Germany) with 160°C oven temperature and nitrogen 5.0 as a carrier gas [20]. For methane, the limit of detection (LOD) was 1 ppm. Calibration was performed using a gas mixture composed of methane 5.0 (1%), carbon dioxide 5.0 (20%) and hydrogen (5%) in nitrogen 5.0 atmosphere (Messer, Austria). Different injection volumes were used for calibration (3 point). Samples for GC measurements were taken from small-scale biofilters in an interval of 15 min. For this purpose, 1 mL of gas sample before and after the biofilters, respectively, were analysed for the difference in CH_4_ concentration. Sampling valves were installed in line approx. 3 cm before and 4 cm after the filter. During measurements the biofilters’ exhaust lines were closed via valves after approx. 15 cm to avoid dilution of exhaust gas with ambient air.

### 2.6 16S V4 and ITS3/4 amplicon sequencing

Small-scale biofilters at the end of the investigation period and fresh bark mulch (biofilter seeding material) were subjected to an amplicon sequencing approach. DNA was extracted in triplicates using NucleoSpin® Soil Extract II Kit (MACHEREY&NAGEL, Germany) according to manufacturer’s recommendations, but applying 1 mL of extract as described for the plate counting method: 1 mL of extract was centrifuged at 12 000 x g in the kit’s extraction tubes including beats, subsequently 0.75 mL of supernatant discarded and further proceeded according to the manufacturer’s protocol. Buffer SL1 was used as lysis buffer and elution occurred in 50 μL. The DNA was quantified fluorometrically using Quant-iT™ PicoGreen™ dsDNA Assay Kit (Invitrogen, USA) and an Anthos-Zenyth Multimode Detector [21] and diluted in TrisHCl (pH 8) buffer to obtain a concentration 5 ng DNA/µl. Libraries were prepared in-house according to well established protocols as described previously [18] targeting the V4 region for procaryotes and the ITS region for fungi [22]. A Q5® Hot Start High-Fidelity DNA Polymerase Master Mix (NEB, Germany), 20% enhancer (NEB, Germany) and 500 nM of primer pair 515f / 806r [23] for prokaryotes and primer pair ITS3/ITS4 for fungi were used to amplify 5 ng template DNA. The ZymoBIOMICS™ Microbial Community DNA standard was used as MOCK communities. For procession of 16S reads, we used the *mothur* pipeline published previously [18] which is based on the MiSeqSOP of *mothur* (https://mothur.org/wiki/miseq_sop/) which includes preparing a contigs file, quality filtering, alignment, pre-clustering, chimera checks, sequence classification via the k-nearest neighbour algorithm (knn), assigning to operational taxonomic units (OTUs) according to their taxonomy, rarefaction analyses, and subsampling with following modifications: We used the latest mothur version 1.48.2 (RRID: SCR_011947). After quality filtering (*maxhomop=10, maxambig=0, minlength=290, maxlength=300)*, 611 642 16S sequences (about 55%) remained for downstream analyses. Unique sequences were aligned to the SILVA v138.2 [24]. Erroneous (*pre.cluster*) and chimeric (VSEARCH 2.16.0) amplicons were removed in a second quality check (89% remained), and the sequences were classified with the k-nearest neighbour algorithm and SILVA taxonomy. Samples were normalized to 21 260 reads per sample according to rarefaction analyses. Thereby one bark mulch samples did not fulfil the sequencing depth and had to be removed. All prokaryotes of the MOCK community could be recovered correctly. Fungal reads were quality filtered with a minlength of 295 and maxlength of 295 bases (975 503 ITS sequences - about 78% - remained), and subsequently pre-clustered and chimera-checked (97% remained) according to the 16S pipeline. We classified sequences based on the pipeline *mothur.fungal.batch* (https://github.com/krmaas/bioinformatics/blob/master/mothur.fungal.batch) using the UNITE database v10 [25] and *wang* method. We assigned sequences to OTUs based on their taxonomy (command *phylotype, label=2*) and gathered representative sequences for each OTU (command *get.oturep, label=2*). Samples were normalized to 34 520 reads per sample according to rarefaction analyses. All fungal genera of the MOCK community could be recovered correctly.

### 2.7 Data procession and statistical analyses

Data and statistical analysis as well as graphical processing were done using SigmaPlot 15 (Inpixon) and Statistica 13.5 (Tibco). Statistical analyses as well as graphical presentations of metagenomic data were done with RStudio© 2024.09.1 and R version 4.4.2 using the packages *rstatix* [26], *ggpubr* [27] as well as *phyloseq* [28], *ade4* [29], *vegan* [30], *plyr* [31], *knitr* [32], *extrafont* [33], *readxl* [34] and *ggplot2* [35]. After checking homogeneity of variance with the *Levene* test, we used *Tukey’s Honest Significant Difference (HSD)* test for alpha diversity analyses (Evenness, Observed, Shannon). For procaryotic communities, we created a heatmap with *readxl, ComplexHeatmap* [36], and *extrafont*. The stacked bar plot for fungi was done with *ggplot2* [35], *tidyverse* [37], *microbiome* [38], *extrafont, randomcoloR* [39], *dplyr* [40], *phyloseq, readxl*, and *ggeasy* [41]. The core microbiome was analysed using *mothur* command *get.coremicrobiome*.

## 3 Results and discussion

### 3.1 Large-scale biofilter

**Fig. 3:**
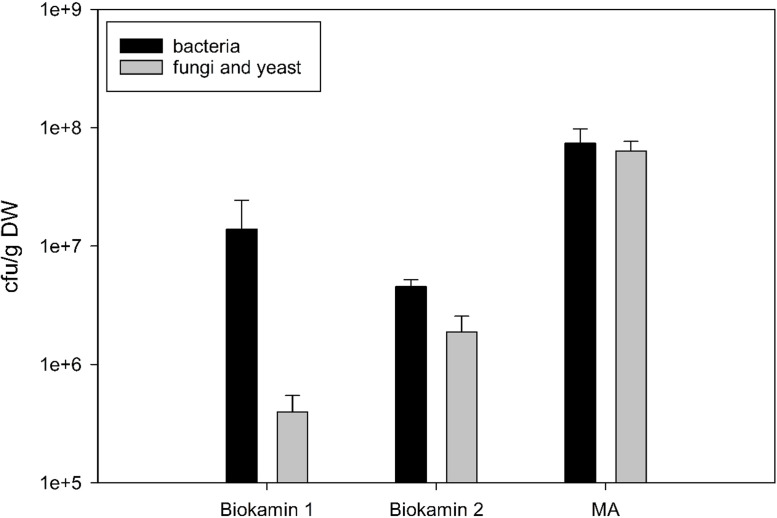
Colonisation [cfu g-1 (DW)] of biofilters composed of bark mulch (Biokamin 1 and 2) and coconut fibre (MA) with bacteria and fungi/yeast, respectively.

A total number of 1.39 × 10^7^ cfu g^-1^ (DW) ± 1.05 × 10^7^ and 4.00 × 10^7^ cfu g^-1^ (DW) ± 4.08 × 10^7^ in bark mulch filter material of Biokamin 1 and Biokamin 2 (Fig. 3), respectively, was found. Biofilter material from coconut fibre (MA) showed the highest numbers with a mean of 7.40 × 10^7^ cfu g^-1^ (DW) ± 2.36 × 10^7^ for total bacteria.

Regarding fungal/yeast growth, the highest numbers were found for coconut fibre (water content of 85.5%) with 6.39 × 10^7^ cfu g^-1^ (DW) ± 1.29 × 10^7^. Bark mulch biofilters showed lower fungal / yeast numbers: 3.98 × 10^5^ cfu g^-1^ (DW) ± 1.47 × 10^5^ for Biokamin 1 and 2.34 × 10^6^ cfu g^-1^ (DW) ± 1.19 × 10^6^ for Biokamin 2. Interestingly, both bark mulch filters showed similar microbial colonisation but exhibited different water contents of 95.5 and 30.4%, respectively, during lab analyses. Bacterial growth was merely affected by the rather low water contents, whereas total numbers of yeast and fungi were clearly reduced at higher water contents of > 85 g 100 g^-1^, which would be generally preferred by operators.

Using bark mulch or coconut fibre as a biofilter material provides the necessary environment to allow growth of procaryotes and fungi/yeast. Additional inoculation was not done by the operator of biofilters at the biogas plant facility. Attributing positive or negative odour removing characteristics to a certain number of microbes is, however, challenging because of a general lack of reference data. However, it must be stated here again, that the odour removal performance of the tested biofilters was not perfect when samples were taken (personal communication biofilter operators) – irrespective of the biofilter and cfu counts.

### 3.2 Small-scale biofilter

**Fig. 4:**
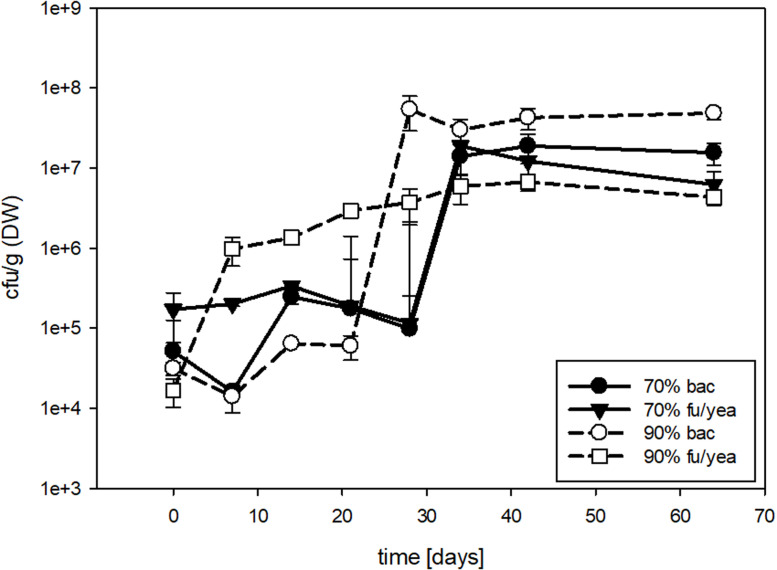
Number of cfu for bacteria (bac) and fungi/yeast (fu/yea) in small-scale bark mulch biofilters with 70 and 90% water content, respectively, during an investigation period of 64 days.

Small-scale biofilters (run in lab experiments) adjusted to a water content of 70 and 90%, respectively, were sampled over a period of 64 days and analysed for the total number of cultivable microorganisms (cfu, Fig. 4). While numbers for bacteria and fungi/yeast in biofilter material with a 70% water content did not change dramatically within the first 4 weeks and both numbers stayed in the range of approx. 1 × 10^5^ cfu g^-1^ (DW)), significant increase of approx. 2 powers of ten was observed for both filters (Fig. 4) thereon. In biofilter samples with 90% water content, similar to the 70% biofilters, bacterial counts remained constant for 3 weeks but then increased dramatically and were constant (approx. 5 × 10^7^ cfu g^-1^ (DW)) after that. By contrast, fungi/yeast numbers increased steadily but stabilised at a lower level of approx. 5 × 10^6^ cfu g^-1^ (DW) (Fig. 4). Consequently, stabilisation of cfu numbers were achieved after 30 to 40 days reflecting a kind of steady state within the biofilters, independently of the water content.

Cfu counts were comparable with large-scale biofilters as both systems showed values between 1 × 10^6^ and 1 × 10^8^ cfu g^-1^ (DW). While large-scale biofilters were not operating at full performance and problems with odour nuisance deriving from an unstable large-scale biofilter operation capability were reported (personal communication biogas plant operators) and the extent of microbial colonisation of biofilters did not necessarily provide information on biofilter performance, small-scale biofilters were considered to work well. Exhaust gases of lab-scale biogas reactors were considered odorous via individual tests applying a scale from 0 to 5 with values between 4.00 and 4.67. After passage through the small scale biofilters, the odour nuisance was reduced to a range of 0.67 to 0.83 within the first 14 days and to below 0.5 afterwards, when microbial abundances also increased (Fig. 4). However, further investigations applying certified olfactometry are necessary to gain deeper insights into a possible causal relationship. Nevertheless, it could be demonstrated that the applied, very simple small-scale biofilters successfully reduced odour nuisance and established stable microbial communities after 4 weeks of operation with regard to microbial abundance.

#### 3.2.1 16S V4 and ITS amplicon sequencing of small-scale biofilter material

**Fig. 5:**
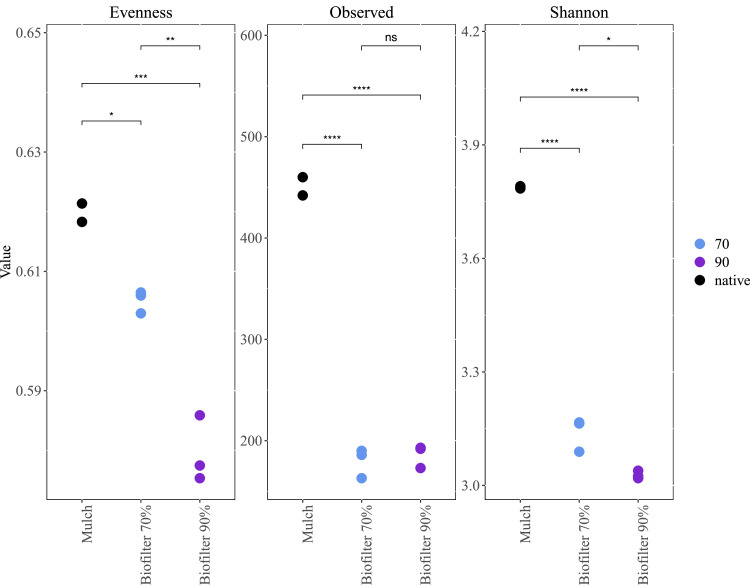
Prokaryotic diversity in small scale biofilters (mulch: bark mulch, biofilters with 70% water content, biofilters with 90% water content).

Prokaryotic diversity and taxon evenness was significantly higher in biofilter starting material (bark mulch) than in biofilter samples (Fig. 5). This indicates that specialised microbial communities evolved during exposure to exhaust biogas, especially at high water content (90%). Despite these differences, several genera like *Acidothermus* were abundant in all samples (core microbiome of starting material and biofilter samples, Table 2). Many species of those core genera are acidophilic which is in accordance with the acidic nature of bark mulch [42]. So far, *A. cellulolyticus* is the only described species and known for its broad spectrum of biomass degrading enzymes [43] and has been previously found in soil samples according to the Microbe Atlas Project [44]. *Silvibacterium* spp. is also a typical genus found in coniferous forests, which was also found to be in the core microbiome of the biofilter samples (e.g., *S. bohemicum*) [45].

**Table 2:** Core microbiome of all bark mulch and biofilters for prokaryotes and fungi.

Core microbiome *Bacteria*	Core microbiome *Fungi*
*Acidothermus spp.*	*Paecilomyces spp.*
*Alicyclobacillus spp.*	*Pleurostoma spp.*
*Granulicella spp.*	*Aspergillus spp.*
*Silvibacterium spp.*	*Talaromyces spp.*

**Fig. 6:**
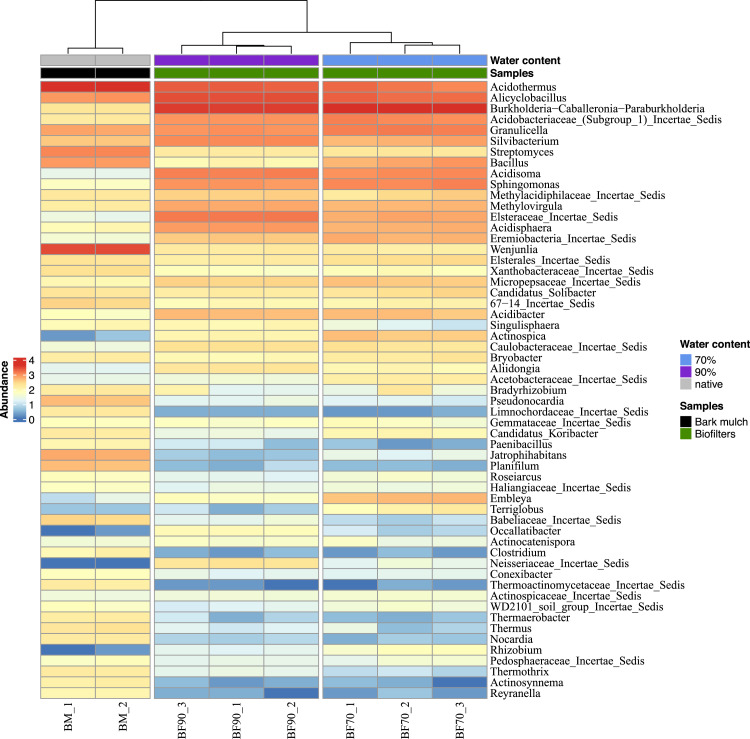
Heat map of prokaryotic diversity and relative abundance in in small scale biofilters (70%: biofilters with 70% water content, 90%: biofilters with 90% water content) in comparison to starting material (native: bark mulch).

Moreover, methane concentration was regularly measured in empty biofilters (before bark mulch addition) for a period of 3 weeks before the actual biofilter experiment started. We measured methane concentrations of 55.8% (± 1.36) and 54.0% (± 1.70) before and after biofilters, respectively. In the course of the actual biofilter experiment, after 28 days, a methane reduction of 6.7% (vol) was observed in biofilters with 70% water content after bark mulch passage (Fig. 2). This reduction was either due to physico-chemical effects on the biofilter’s matrix (e.g. adsorption) or to methanotrophy. However, biofilters with 90% water content did not show any reduction in CH_4_ concentrations. This is plausible as a high-water content reduces oxygen availability which is a prerequisite for methanotrophy performed by aerobic microorganisms. Aerobic methanotrophy is widespread within the phyla *Proteobacteria, Verrucomicrobia* [46] and – as quite recently observed – also in *Actinobacteria* (highly acidic environments) [47]. In the present study, the relative abundance of *Methylovirgula* spp. was significantly higher in biofilters than in control samples, indicating an enhanced growth in the presence of exhaust biogas (Fig. 6). *Methylovirgula* species are mesophilic, obligate acidophilic methylotrophs and *Methylovirgula thiovorans* is the only described *Methylovirgula* species to be methanotrophic and the only described methanotroph to be capable of oxidising methane and thiosulfate simultaneously or independently [48]. Another abundant methanotroph in biofilter samples was an unknown species of the extremophile family *Methylacidiphilaceae* (Fig. 6). *Methylacidophilum fumariolicum* SolV was shown to simultaneously oxidise methane and hydrogen sulfide. It was postulated that this feature is more common in methanotrophs than expected, and important to overcome sulfide toxicity during methanotrophy [46]. Regarding the methane-rich environment of the biofilters and the considerable reduction of odour nuisance, it is plausible that *Methylovirgula and Methylacidiphilaceae* species were active in biofilters and oxidised methane and/or sulphur compounds. As both taxa are metabolically versatile, it is conceivable that they thrive also in methane-free (but acidic) environments such as bark mulch.

**Fig. 7:**
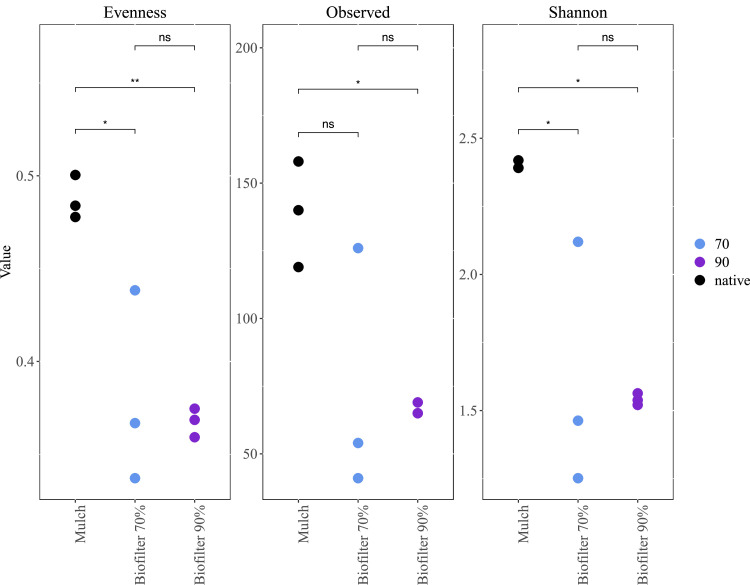
Fungal diversity in small scale biofilters (mulch: bark mulch, biofilters with 70% water content, biofilters with 90% water content).

**Fig. 8:**
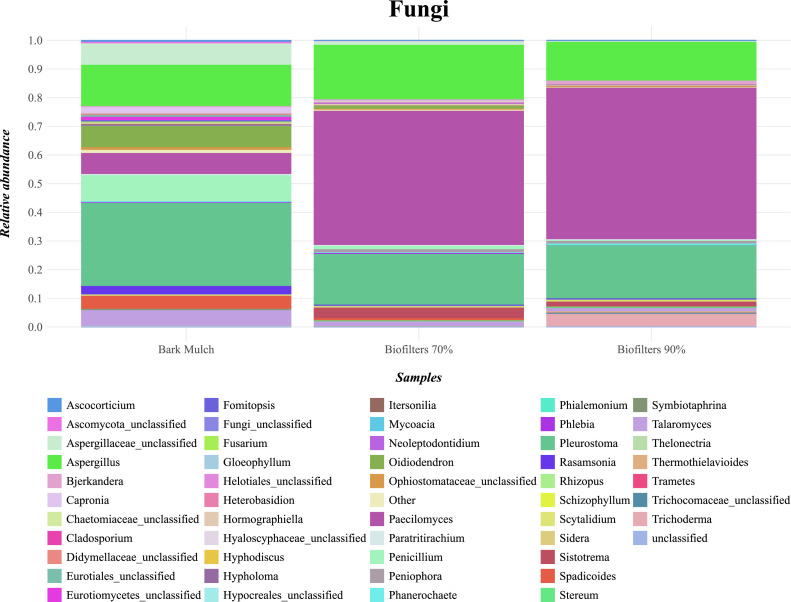
Stacked bar plot of fungal community composition (ITS3/4 amplicon sequencing) in small scale biofilters (bark mulch, biofilters with 70% water content, biofilters with 90% water content).

Comparable to prokaryotic data, fungal diversity and species evenness was significantly higher in bark mulch than in biofilter samples (Fig. 7). The relative abundance of *Paecilomyces* spp., a core microorganism in all variants, increased in biofilter samples – irrespective of the water content (Fig. 8). This fungal genus produces various bioactive, secondary metabolites with an exceptional potential for biotechnological applications such as pest control [49]. A decrease in relative abundance for the genus *Oidiodendron* was found in the biofilters when compared to the bark mulch samples. This might be due to the fact that many representatives of this genus show reduced growth at temperatures at 25°C [50] and biogas exhaust temperature was >25°C when entering the biofilters due to thermophilic operation conditions of biogas reactors. However, also an increase in rather undesired organisms like *Aspergillus* sp was found (Fig. 8).

## 4 Conclusions

Large-scale biofilters showed a similar total abundance of bacteria irrespective of the filter material (bark mulch or coconut fibre) and the water content of the biofilter, while fungal and yeast abundance was impacted by both factors. In small scale biofilters (composed of bark mulch), the water content impacted microbial abundance and methane reduction potential (biotic and/or physico-chemical). While a water content of 90% led to a similar development of bacterial and fungal/yeast abundance, 70% water content caused an asynchronous increase in abundance. Generally, the diversity and taxon evenness were significantly higher in biofilter starting material (bark mulch) than biofilter samples, indicating that specialised eu- and prokaryotic communities evolved during exposure to exhaust biogas. The biofilter communities comprised several acidotrophic bacteria including potential methanothrophs and (thio)sulfate devouring bacteria. Indeed, small-scale biofilters efficiently removed odorous compounds from biogas, especially when pro- and eukaryotic abundances reached 10^6^ cfu g^-1^ DW, and biofilters with 70% water content showed slightly reduced methane concentrations after bark mulch passage. However, abundance data *per se* did not allow conclusions on biofilter performance. Further studies are required to better understand the relation between biofilter performance and microbial abundance and whether the reduction of methane can be contributed to methanotrophy or physico-chemical effects.

## Acknowledgements

We greatly thank ABV Roppen, Westtirol, for supporting this study.

## Data statement

Data are available on request via corresponding author.

## Funding sources

The study was supported by the Austrian Science Fund (FWF: P36711, Grant DOI 10.55776/P36711) project P33838, Grant DOI 10.55776/P33838 and ESP 7170024, Grant DOI 10.55776/ESP7170024.

## CRediT authorship contribution statement

**Andreas Otto Wagner:** Writing – original draft, Visualization, Supervision, Resources, Project administration, Methodology, Funding acquisition, Formal analysis, Data curation, Conceptualization. **Julia Wurm:** Writing – review & editing, Investigation, Data curation. **Mathias Wunderer:** Writing – review & editing, Supervision, Data curation. **Julia Zöhrer:** Writing – review & editing, Supervision, Data curation. **Andja Mullaymeri:** Writing – review & editing, Supervision, Data curation. **Eva-Maria Weinseisen:** Writing – review & editing, Resources. **Eva Maria Prem:** Writing – review & editing, Visualization, Validation, Project administration, Data curation, Conceptualization.

## Declaration of competing interest

The authors declare the following financial interests/personal relationships which may be considered as potential competing interests:

Andreas Otto Wagner reports financial support was provided by Austrian Science Fund. If there are other authors, they declare that they have no known competing financial interests or personal relationships that could have appeared to influence the work reported in this paper.

## Data Availability

Sequences of metagenomic analyses have been submitted to the European Nucleotide Archive (ENA) under the study accession number PRJEB90291.
